# Improved estimation of aboveground biomass in wheat from RGB imagery and point cloud data acquired with a low-cost unmanned aerial vehicle system

**DOI:** 10.1186/s13007-019-0402-3

**Published:** 2019-02-20

**Authors:** Ning Lu, Jie Zhou, Zixu Han, Dong Li, Qiang Cao, Xia Yao, Yongchao Tian, Yan Zhu, Weixing Cao, Tao Cheng

**Affiliations:** National Engineering and Technology Center for Information Agriculture (NETCIA), Key Laboratory for Crop System Analysis and Decision Making, Ministry of Agriculture and Rural Affairs, Jiangsu Key Laboratory for Information Agriculture, Jiangsu Collaborative Innovation Center for Modern Crop Production, Nanjing Agricultural University, One Weigang, Nanjing, 210095 Jiangsu China

**Keywords:** Aboveground biomass, UAV, Canopy height, Spectral vegetation index, RGB camera

## Abstract

**Background:**

Aboveground biomass (AGB) is a widely used agronomic parameter for characterizing crop growth status and predicting grain yield. The rapid and accurate estimation of AGB in a non-destructive way is useful for making informed decisions on precision crop management. Previous studies have investigated vegetation indices (VIs) and canopy height metrics derived from Unmanned Aerial Vehicle (UAV) data to estimate the AGB of various crops. However, the input variables were derived either from one type of data or from different sensors on board UAVs. Whether the combination of VIs and canopy height metrics derived from a single low-cost UAV system can improve the AGB estimation accuracy remains unclear. This study used a low-cost UAV system to acquire imagery at 30 m flight altitude at critical growth stages of wheat in Rugao of eastern China. The experiments were conducted in 2016 and 2017 and involved 36 field plots representing variations in cultivar, nitrogen fertilization level and sowing density. We evaluated the performance of VIs, canopy height metrics and their combination for AGB estimation in wheat with the stepwise multiple linear regression (SMLR) and three types of machine learning algorithms (support vector regression, SVR; extreme learning machine, ELM; random forest, RF).

**Results:**

Our results demonstrated that the combination of VIs and canopy height metrics improved the estimation accuracy for AGB of wheat over the use of VIs or canopy height metrics alone. Specifically, RF performed the best among the SMLR and three machine learning algorithms regardless of using all the original variables or selected variables by the SMLR. The best accuracy (*R*^2^ = 0.78, RMSE = 1.34 t/ha, rRMSE = 28.98%) was obtained when applying RF to the combination of VIs and canopy height metrics.

**Conclusions:**

Our findings implied that an inexpensive approach consisting of the RF algorithm and the combination of RGB imagery and point cloud data derived from a low-cost UAV system at the consumer-grade level can be used to improve the accuracy of AGB estimation and have potential in the practical applications in the rapid estimation of other growth parameters.

## Background

Aboveground biomass (AGB) is a critical indicator in crop growth status monitoring and grain yield prediction [1]. Accurate and rapid estimation of AGB is crucial for the assessment of crop nutrition status and the improvement of crop management strategies. The conventional estimation of AGB is based on destructive measurements [2], which are not only time consuming and labor intensive, but also hard to apply over large areas [3]. Remote sensing as a non-destructive technique has been proved to have great potential in AGB estimation for crops, such as wheat [4, 5], barley [6], maize [7] and rice [8].

The majority of previous studies on the remote estimation of AGB focused on the use of remotely sensed data acquired from ground [4, 8], man-made aircraft [9] and satellite platforms [10]. For instance, Cheng et al. [8] reported a *R*^2^ up to 0.81 for the relationship between the red-edge chlorophyll index (CI_Red-edge_) and rice biomass using ground-based hyperspectral data. Although ground-based remote sensing can yield satisfactory estimation accuracy for crop growth parameters, they are costly to acquire and unsuitable for monitoring over large areas [11]. In contrast, the satellite platform has great advantages in acquiring crop growth information over regional and large scales. As reported by Wang et al. [12], a high accuracy (*R*^2^ = 0.79) could be obtained from HJ-1 satellite imagery for the estimation of wheat AGB at the anthesis stage over four counties of Jiangsu province, China. However, as the growth status of crop varies rapidly across critical growth stages, multi-temporal and timely acquisition of remotely sensed data is necessary for crop monitoring [7]. It is challenging to acquire suitable satellite imagery for monitoring over multiple growth stages due to the frequent cloud cover and the inadequate spatial resolution matching relatively small field sizes in China, especially the lower reaches of Yangtze River [13]. Using manned airborne platforms may overcome these limitations and acquire images with high temporal and spatial resolutions, but it is often complex and costly to allocate aircraft and instrument resources.

The advent of unmanned aerial vehicles (UAVs) makes it possible to acquire high temporal and spatial resolution remotely sensed data in an affordable way. In recent years, multiple types of cameras for acquiring RGB, color-infrared (CIR), multispectral, and hyperspectral images have been mounted on various UAV platforms for monitoring crop growth status [14–16]. Particularly, much attention has been paid to low-cost UAV systems consisting of RGB or modified CIR cameras and light-weight drones. The low-cost UAV systems were widely used due to the most significant advantages in affordability, ease of operation, and simplicity in image processing [11, 17–19]. People often use the visible or CIR images collected with these low-cost UAV systems to generate orthophotos and point cloud data for crop growth monitoring. While the former type of data could be used to extract vegetation indices (VIs) for estimating crop biophysical and biochemical parameters with moderate accuracies, such as biomass [1, 20], the latter could be used to construct crop surface models (CSMs) for estimating crop structural parameters with high accuracies, such as plant height (PH) [21, 22].

Previous studies have demonstrated that both VIs and canopy height metrics (e.g., height percentiles) derived from UAV images are critical variables for estimating crop biomass [1, 7]. However, the majority of those studies utilized either VIs [19, 23] or canopy height metrics alone [21] for model establishment [24]. While the VIs composed of visible or CIR bands characterized the spectral properties of the top canopy, the height metrics reflected the vertical structure properties of the entire canopy. Although these two types of variables were used to extract different sources of information about the crop canopy, the performance of either type of variables for biomass estimation might be limited by the insensitivity of VIs at high biomass conditions and the stability of plant height at reproductive stages [1, 25, 26]. The estimation of crop biomass from UAV images might be improved by using the two complementary data sources simultaneously.

In recent years, there are some attempts to improve the estimation of crop biomass by combining VIs and canopy height metrics (Table 1). For instance, Bendig et al. [6] combined the GnyLi index derived from ground-based hyperspectral data and canopy height metrics acquired from a low-cost UAV system to obtain a *R*^2^ of 0.82 for barley biomass estimation. Tilly et al. [1] also achieved a high accuracy with the fusion of ground-based hyperspectral data and canopy height acquired from terrestrial LiDAR data. Nevertheless, these studies focused on the combination of VIs and canopy height metrics derived from two different sensors, which may limit the applications over large areas due to the high cost of an expensive sensor. In contrast, Li et al. [7] reported the fusion of VIs and canopy height metrics acquired from a low-cost UAV system for the estimation of maize biomass with three regression techniques (simple linear regression, stepwise linear regression and random forest regression), but they did not explicitly compare the performances among VIs, canopy height metrics and their combination. Therefore, it remains unclear whether the combined data generated from a single sensor could lead to improved estimation of biomass without any additional cost in instrumentation. In addition, their data only cover one growth stage of maize and they are inadequate for assessing the performance of those input variables over all critical growth stages.

**Table 1:** Summary of published studies on the estimation of plant height and biomass of crops from RGB imagery acquired from unmanned aerial vehicles (UAVs)

Reference	Crop type	UAV	Sensors	Regression method	VIs/SfM	Canopy characteristic	Best accuracy
Bendig et al. [21]	Barley	MK-Oktokopter	Panasonic Lumix GX1 (RGB)	ER	–	Biomass	*R*^2^ = 0.82
						PH	*R*^2^ = 0.92
Watanabe et al. [27]	Sorghum	USM-S1	Powershot ELPH 110HS	GPM	–	PH	*r *= 0.84
Schirrmann et al. [28]	Wheat	Hexacopter (P-Y6)	Sony Nex 7	LR	–	Biomass	*r* = 0.68
				PCA		PH	*r* > 0.80
Iqbal et al. [22]	Poppy	Oktokopter	Canon 550D DSLR	LR	SfM	PH	*R*^2^ = 0.71
						Volume	*R*^2^ = 0.71
Roth et al. [17]	Winter wheat	ARF Mikrokopter Okto XL	Canon EOS 100D	LR	None	Biomass	*R*^2^ = 0.74
						PH	*R*^2^ = 0.80–0.84
						CC	
Bendig et al. [6]	Barley	MK-Oktokopter	Panasonic Lumix GX1 (RGB) + FieldSpec3	MLR	VIs + SfM	Biomass	*R*^2^ = 0.84
				MNLR			
Kim et al. [29]	Cabbage	DJI F550 Hexa-rotor	Powershot S110 RGB	MLR	–	PH	*R*^2^ > 0.90
Li et al. [7]	Maize	Rotor-wing UAV	Sony A6000	MSLR	VIs + SfM	Biomass	*R*^2^ = 0.78
				RF		PH	*R*^2^ = 0.88
Holman et al. [30]	Wheat	DJI Wookong M	Sony Nex 7	–	SfM	PH	RMSE = 3.00 cm
						Growth rate	
Madec et al. [31]	Wheat	Hexacopter	Sony ILCE-6000	–	SfM	PH	RMSE = 3.50 cm

*CC* Canopy cover, *ER* exponent regression, *GPM* genomic prediction modeling, *LR* linear regression, *MLR* multiple linear regression, *MNLR* multiple non-linear regression, *MSLR* multiple stepwise linear regression, *PCA* principal components analysis, *PH* plant height, *RF* random forest, *SfM* structure from motion, *VIs* vegetation indices

For the use of the comprehensive information in multiple types of remotely sensed data, multivariate regression techniques could be an essential approach for establishing direct relationships between remotely sensed variables and crop parameters [32], including multiple linear regression (MLR) [33], stepwise multiple linear regression (SMLR) [34] and partial least squares regression (PLSR) [5]. However, these regression techniques are more suitable for the data that exhibit linear or exponential relationships between remotely sensed variables and crop biophysical/biochemical parameters [35, 36]. Moreover, the VIs and canopy height metrics derived from a consumer-grade camera may be redundant and highly autocorrelated. In contrast to conventional regression techniques, machine learning regression algorithms such as random forest (RF) [37], support vector regression (SVR) [38] and extreme learning machine (ELM) [39] are typically better at handling high-dimensional data and the non-linear relationships [40, 41]. Recent studies have also found that machine learning algorithms could yield higher accuracies for biomass estimation than conventional ones [7, 42].

To the best of our knowledge, few studies have examined machine learning techniques for the estimation of wheat AGB by combining the canopy spectral information and vertical structure information from UAV-derived VIs and canopy height metrics. Since such combined data could be obtained from a RGB camera on board a small UAV, it becomes necessary to investigate the performance of a consumer-grade level UAV system at an even lower cost. Thus, the objectives of this study were: (1) to examine the feasibility of combining spectral indices and canopy height metrics from a RGB camera mounted on a consumer-grade UAV for the improved estimation of AGB in wheat; (2) to evaluate the performance of three machine learning regression techniques over critical growth stages and spatial resolutions in comparison to the traditional SMLR.

## Methods

### Experimental design

**Fig. 1:**
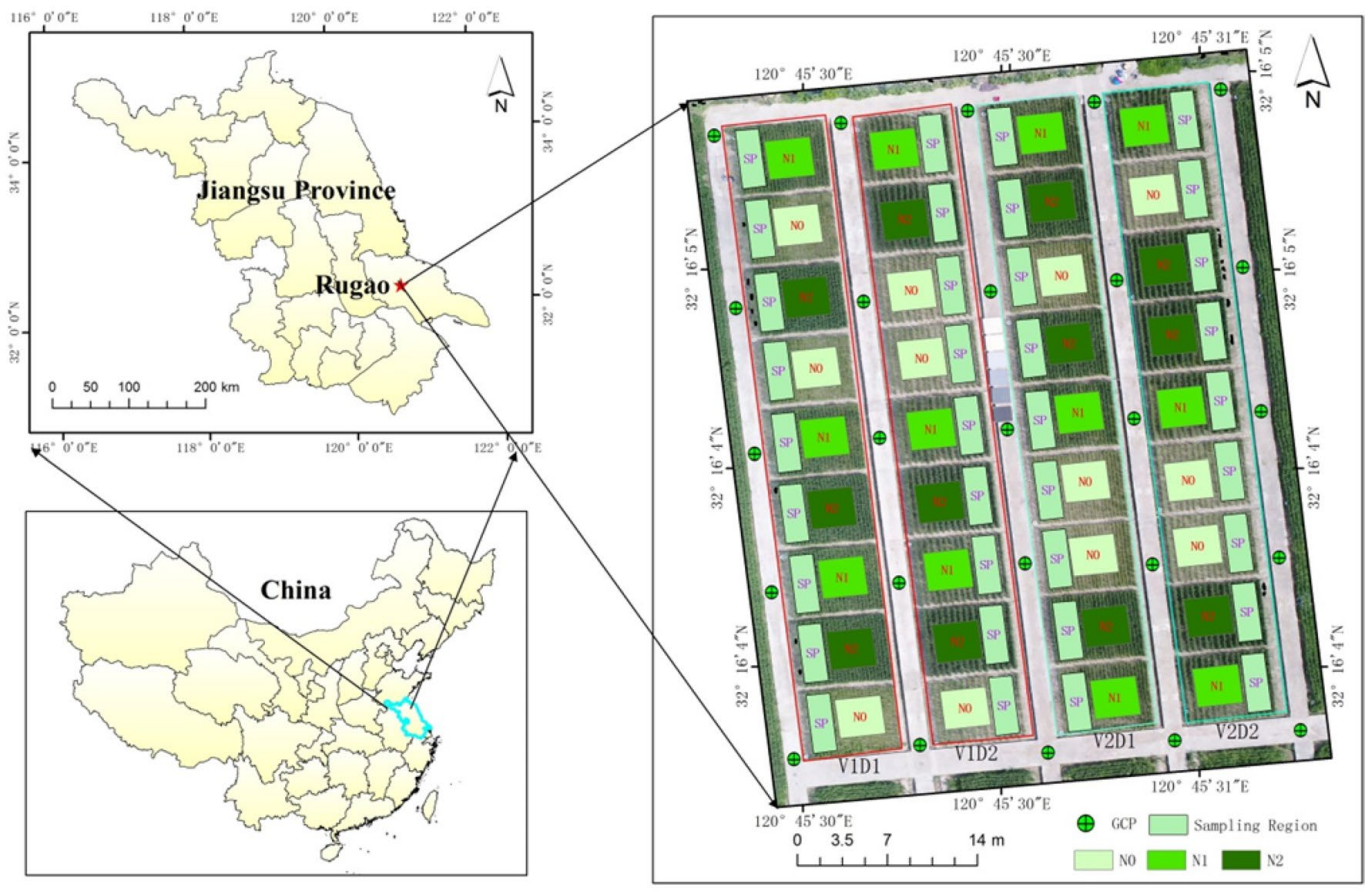
Location of the experimental site and layout of the field plots randomly distributed with treatments in nitrogen level, wheat variety and sowing density. The orthophoto on the right was captured with the UAV system at the anthesis stage of wheat on April 22, 2017. *Note*: *GCP* ground control point, *SP* sampling region; D1 = 0.25 m, D2 = 0.4 m, N0 = 0 kg N ha^−1^, N1 = 150 kg N ha^−1^, N2 = 300 kg N ha^−1^, V1 = ‘Yangmai 15’, V2 = ‘Yangmai 16’

Two experiments were conducted in the experimental station of the National Engineering and Technology Center for Information Agriculture (NETCIA) located in Rugao, Jiangsu province of eastern China (120º45′E, 32º16′N) (Fig. 1). A total of 36 plots were used for the experiments spanning two wheat growing seasons. The plots with a size of 6 × 5 m^2^ covered different wheat cultivars, planting densities and nitrogen (N) rates. In order to avoid the complexity of soil N levels, we applied the same N level as that in the preceding rice growing season for each plot. The detail of experimental design can be found as follows.

Experiment 1 was conducted in the winter wheat season of 2015–2016 with the sowing date of October 30, 2015. One wheat cultivar with the erectophile leaf type, ‘Yangmai 18’, was used for all plots. Four N rates (0, 80, 150, 220 kg N ha^−1^) and three planting densities (2.0 × 10^6^ plants ha^−1^, 1.3 × 10^6^ plants ha^−1^ and 1.0 × 10^6^ plants ha^−1^, corresponding to 0.2 m, 0.3 m and 0.4 m row spacings) were applied with three replications. These N levels and planting densities could cover the possible rates used in local agronomic practices and lead to variations in AGB, canopy cover and background materials between plots. The N fertilizers were applied in 50% as basal fertilizer at the sowing day and 50% at the jointing stage.

Experiment 2 was conducted in the winter wheat season of 2016–2017 with the sowing date of November 15, 2016. Two winter wheat cultivars with different canopy structures, ‘Yangmai 15’ and ‘Yangmai 16’, were selected to represent planophile and erectophile leaf types, respectively. Three N rates (0, 150, 300 kg N ha^−1^) with two planting densities (1.6 × 10^6^ plants ha^−1^ and 1 × 10^6^ plants ha^−1^, corresponding to 0.25 m and 0.4 m row spacings) were applied with three replications. 50% of N fertilizers were applied at the sowing day and 50% at the jointing stage.

### Data collection

The UAV system for image collection was the DJI Phantom series (Edition 3 in 2015 and Edition 4 in 2016 with added obstacle avoidance for flight safety and slight upgrade in camera specifications as shown in Table 2), both of which represent a low-cost UAV system consisting of a four-rotor drone and a digital camera (SZ DJI Technology Co., Shenzhen, China). Before the initial flight, we set 25 ground control points (GCPs) with marked signs on the concrete roads across the study site to georeference the UAV images from different growth stages. The geographic coordinates were obtained from RTK-GPS (Real-Time Kinematic Global Positioning System, CHC X900 GNSS) with horizontal and vertical errors within 1 cm and 2 cm, respectively. In our campaigns, the UAV was set to automatic flying mode and followed pre-defined flight plan to acquire imagery with approximately 80% forward overlapping and 60% side overlapping. The images were captured in an automatic mode at 1 frame per 5 s with the JPEG format. The ISO of camera was set to 100 and the best exposure was set based on the weather condition. The aperture of camera was the default with f/5. The same flight path and camera setup excluding exposure time were applied to the whole season. The UAV was flown over the study site at critical growth stages (Table 3) at the height of 30 m above ground level. The speed of UAV was set at 0.5 m/s and it took about 12 min cover the whole study area. Each flight campaign was carried out at 11:00 am–14:00 pm local time during sunny day and acquired approximately 58 images with a spatial resolution of 1.66 cm. In order to generate digital terrain model (DTM) of the study site, an extra flight campaign was conducted after wheat sowing on November 16, 2016.

**Table 2:** Technical specifications of the cameras used in the two consecutive UAV editions for the two wheat seasons

Edition of camera	Field of view	Image size	Focus length	Image format
Phantom 3	94°	4000 * 3000	f/2.8	JPEG; DNG
Phantom 4	84°	4864 * 3648	f/2.8–f/11	JPEG; DNG (RAW); JPEG + DNG

**Table 3:** Summary of field campaigns for the wheat experiments

Experiment	Sowing date	Date of UAV flights	Date of field sampling	Growth stage
#1	October 30, 2015	March 22, 2016	March 22, 2016	Jointing
		April 8, 2016	April 9, 2016	Booting
		April 17, 2016	April 17, 2016	Heading
		April 22, 2016	April 21, 2016	Anthesis
#2	November 15, 2016	November 16, 2016	–	–
		March 17, 2017	March 18, 2017	Jointing
		March 27, 2017	March 28, 2017	Booting
		April 12, 2017	April 12, 2017	Heading
		April 22, 2017	April 22, 2017	Anthesis

Field sampling of AGB from the 36 plots were conducted within 1 day of the UAV campaigns. Since the destructive sampling was conducted four times in each growing season, only a total of 30 plants were randomly harvested from each sampling region in Fig. 1 to represent each of the homogenous plots. The plants from each plot were harvested from above the ground and then separated into leaves, stems and panicles (for post-heading stages only). All components were oven-dried at 105 °C for 30 min and afterwards at 80 °C for about 48 h until a constant weight. The dry biomass of wheat organs (leaves, stems and panicles) was weighted, respectively. Moreover, the number of plants per unit ground area was also counted manually in the experimental fields. The AGB in tons per hectare (t/ha) was determined as the product of the dry weight per sampling plant and the number of plants per area. The basic statistics of the field-measured AGB was shown in Table 4. The plant height was measured with a ruler as the distance from the bottom to the top of wheat canopy. Five plants were randomly selected to represent the canopy height of each plot.

**Table 4:** Basic statistics of the field-measured aboveground biomass (AGB, t/ha) of wheat

Year	Min.	Max.	Mean	SD
2016	0.75	15.88	5.23	2.89
2017	0.62	12.12	3.86	2.60
Pooled	0.62	15.88	4.55	2.82

### Generation of orthophotos and crop surface models

The UAV images were processed within the software Agisoft Photoscan 1.2.6 (Agisoft LLC, St. Petersburg, Russia) to generate orthophotos and digital surface models (DSMs). The key processing steps included image alignment, camera calibration, construction of dense point clouds, and generation of orthophotos and digital elevation models (DEMs). Firstly, the software automatically aligned the overlapping images using a feature point matching algorithm. Secondly, seven of the twenty-five evenly distributed GCPs were used to georeference each image. The camera internal parameters were estimated in Agisoft Photoscan based on image alignment and the GCPs positions. The estimated parameters were then used to compensate a linear model misalignment while georeferencing the model. Since the top of wheat canopy is sharp and small, we chose ‘Mild’ depth filtering recommended for reconstructing small details to build dense point cloud. Lastly, the orthophotos and DEMs used as crop surface models (CSMs) were generated after building mesh and texture with default parameters and exported as a TIFF image format for subsequent analysis. The details of processing steps and parameter settings can be found in Table 5.

**Table 5:** Processing steps with corresponding parameter settings in Agisoft Photoscan software for generation of orthophotos and DEMs from UAV imagery

Task	Parameter setup
Aligning image	Accuracy: high
	Pair selection: generic
	Key points: 40,000
	Tie points: 4000
Building mesh	Surface type: height field
	Source data: dense cloud
	Face count: high
Positioning guided marker	Manual positioning of markers on the even 7 GCPs for all the photos
Optimizing cameras	Default settings
Building dense point cloud	Quality: high
	Depth filtering: mild
Building texture	Mapping mode: Generic
	Blending mode: Mosaic
	Texture size/count: 4096
Building DEM	Surface: Mesh
	Other parameters: default
Building orthomosaic	Surface: Mesh
	Other parameters: default

### Calculation of spectral indices

This study examined ten published VIs for the estimation of wheat AGB (Table 6). Most of the selected VIs have been related to crop biophysical and biochemical parameters, such as LAI [43], vegetation fraction [44], grain yield [45], biomass [1, 7], and nitrogen accumulation [11]. These VIs were directly calculated using digital numbers from the orthophotos. In addition, a region of interest (ROI) was delineated from each plot within the orthophotos using ArcGIS 10.2.2 (Esri, Redlands, CA, USA) to exclude the border effect and the sampling region. The mean VIs of each ROI were extracted to represent the values of each plot.

**Table 6:** Summary of vegetation indices derived from the aerial orthophotos for the estimation of aboveground biomass in wheat

Index	Name	Formulation	References
VARI	Visible Atmospherically Resistant Index	${\text{VARI}} = \frac{g - r}{g + r - b}$	Gitelson et al. [46]
ExG	Excess Green Index	ExG = 2 * *g* − *r* − *b*	Woebbecke et al. [47]
ExR	Excess Red Vegetation Index	${\text{ExR}} = \frac{1.4R - G}{G + R + B}$	Meyer et al. [48]
ExB	Excess Blue Vegetation Index	${\text{ExB}} = \frac{1.4*B - G}{G + R + B}$	Mao et al. [49]
ExGR	Excess Green minus Excess Red	ExGR = ExG–ExR	Neto et al. [50]
GRVI	Green Red Vegetation Index	${\text{GRVI}} = \frac{G - R}{G + R}$	Tucker et al. [51]
MGRVI	Modified Green Red Vegetation Index	${\text{MGRVI}} = \frac{{G^{2} - R^{2} }}{{G^{2} + R^{2} }}$	Bendig et al. [6]
GLI	Green Leaf Index	${\text{GLI}} = \frac{2*g - r - b}{ - r - b}$	Louhaichi et al. [52]
RGBVI	Red Green Blue Vegetation Index	${\text{RGBVI}} = \frac{{G^{2} - B*R}}{{G^{2} + B*R}}$	Bendig et al. [6]
IKAW	Kawashima Index	${\text{IKAW}} = \frac{R - B}{R + B}$	Kawashima et al. [53]

*R*, *G* and *B* represent the digital number of red, green and blue channels, respectively. *r* = *R*/(*R* + *G* + *B*), *g* = *G*/(*R* + *G* + *B*), *b* = *B*/(*R* + *G* + *B*)

### Determination of canopy height metrics

To estimate the AGB of wheat, eight canopy height metrics (mean, median, standard deviation, coefficient of variation and percentiles 25%, 50%, 75%, 95%) were calculated from each canopy height model (CHM) (Table 7). The CHM was determined as the difference between CSM and DTM excluding outliers for each flight survey. The DTM for the entire season was determined from the images acquired during the post-sowing flight on November 16, 2016, while the CSM was derived from the UAV images for each growth stage to reflect crop growth dynamics. The same ROIs used for VI calculation were applied to the CHMs to extract plot-level canopy height metrics within ArcGIS 10.2.2 (Esri, Redlands, CA, USA).

**Table 7:** Summary of canopy height metrics used in this study for the estimation of aboveground biomass in wheat

Input variable	Name	Formulation
H_mean_	Mean height	$H_{mean} = \frac{1}{n}\mathop \sum \limits_{i = 1}^{n} h_{i}$
H_median_	Median height	$H_{median} = median\;of\;h_{i}$
H_percentile_	Percentile height	25%, 50%, 75%, 95%
H_std_	Standard deviation of height	$H_{std} = \sqrt {\frac{1}{n}\mathop \sum \limits_{i = 1}^{n} \left( {h_{i} - H_{mean} } \right)}$
CV	Coefficient of variation	${\text{CV}} = \frac{{H_{std} }}{{H_{mean} }}$

### Regression techniques

Machine learning algorithms are widely used to handle the strong non-linearity between crop biophysical/biochemical parameters and remotely sensed variables. Compared to parametric regression techniques, machine learning algorithms are well suited for establishing predictive models with multiple input variables. To establish individual models for AGB estimation using ten VIs, eight canopy height metrics and their combination, we used three machine learning techniques implemented with the *caret* package in R x64 3.4.0 environment software (R Development Core Team, 2017).

RF is an ensemble learning method that combines a large number of decision trees to improve the accuracy of classification and regression trees (CART) [37]. Each tree is built with a deterministic algorithm by selecting a random set of variables and a random sample from the calibration dataset. RF regression not only handles a large number of input variables, but also obtains a reasonable prediction accuracy using a small subset of variables [10]. In addition, RF regression is beneficial to overcome the over-fitting problem of simple decision trees. For implementation, the two significant parameters (mtry and ntree) need to be optimized to obtain the best predictive power.

ELM is a single-hidden layer feed forward neural network (SLFN), whose learning speed is relatively faster than the conventional feed forward network [39]. ELM is composed of an input layer, a hidden layer, and an output layer. Unlike traditional neural network algorithms, ELM aims to reach the smallest training error and the smallest norm of output weights [54]. The weights of its hidden layer can be randomly generated without iterative optimization, which makes it suitable for real-time training. In addition, ELM is capable of handling complex data and is robust for regressions with multiple highly inter-correlated variables. Its potential in crop monitoring has been demonstrated in a recent study on the estimation of soybean biophysical and biochemical parameters from fused multi-sensor data [42].

SVR is an effective predictive tool based on the statistical learning theory [55]. The advantage of SVR is the ability to handle high-dimensional data and a small number of training samples [38]. Many studies in remote sensing have used SVR to estimate crop biophysical and biochemical parameters [56, 57]. As the critical parameter, kernel function was set as the radial basis function (RBF) to account for the nonlinear relationships in the wheat data.

Since the variables derived from UAV images might be inter-correlated, simple linear regression as measured by Pearson’s correlation coefficients were conducted for the relationships between individual variables and their relationships with AGB. In addition, SMLR was used as the reference for evaluating the performance of the machine learning algorithms relative to traditional techniques.

### Accuracy assessment

Since the goal was to build global models with various regression techniques across multiple treatments, growth stages, and seasons, we pooled the data from 2 years and all growing conditions to form a comprehensive dataset. Global models are more practical than local ones since frequent model calibrations for different growing conditions could be avoided. The pooled dataset was split into two parts with 70% for model calibration and the remainder 30% for model validation. The accuracy of model calibration was evaluated with the coefficient of determination (*R*^2^), the Root Mean Square Error (RMSE) and akaike information criterion (AIC). The estimation accuracies were assessed by the *R*^2^, RMSE and the relative RMSE (rRMSE) with validation data.

## Results

### Determination of wheat canopy height model

**Fig. 2:**
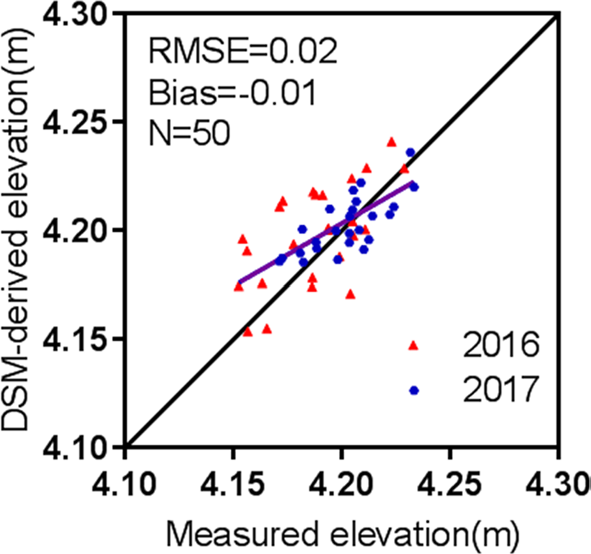
Comparison between measured and DSM-derived elevation for the GCPs throughout the study site

**Fig. 3:**
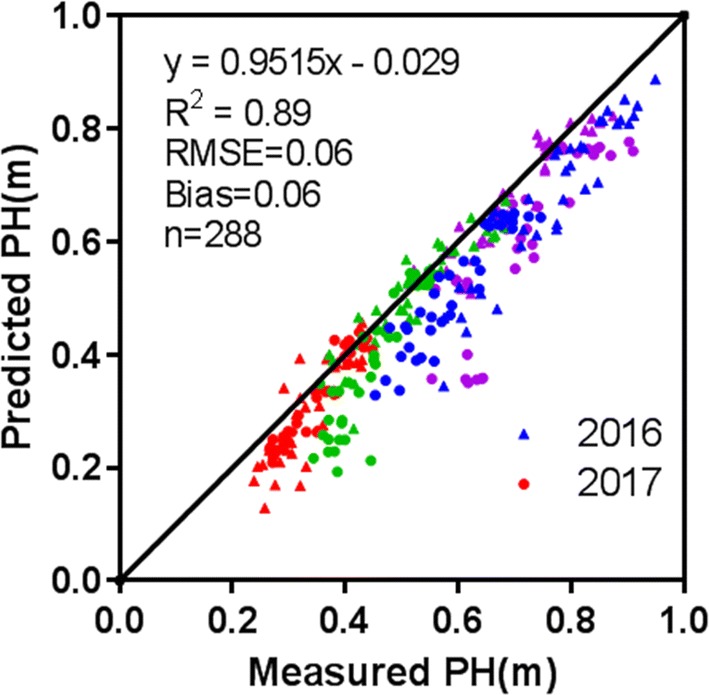
Comparison between measured PH and the PH derived from DSMs generated with UAV images. The diagonal represents the 1:1 line. The data points from 2016 and 2017 are denoted in triangles and solid circles, respectively. Red, green, blue and magenta symbols represent jointing, booting, heading and anthesis stages, respectively

Figure 2 shows a comparison of measured and DSM-derived elevation for the 25 GCPs as an assessment of the DSM elevation accuracy. The RMSE of the GCP elevation estimated with UAV images was 0.02 m for the pooled data from 2016 to 2017. Subsequently, the PH for the field plots derived from the CHM matched well with the field measurements (Fig. 3), exhibiting a *R*^2^ value of 0.89 and a RMSE value of 0.06 m for the 2 years. Overall, the CHM-derived PH was slightly lower than the measured PH (Bias = 0.06 m).

### Correlation between UAV-derived variables

**Fig. 4:**
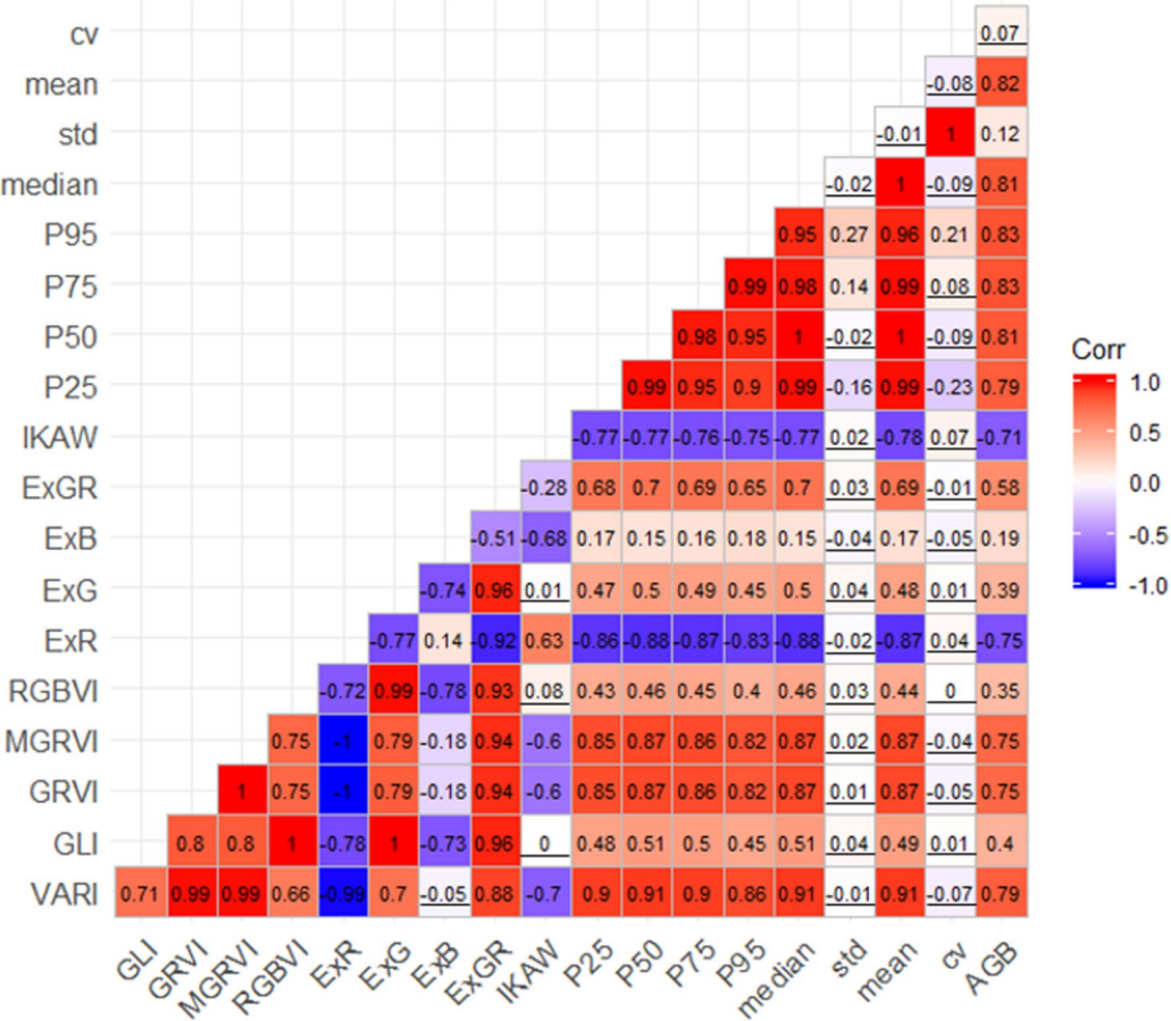
Pearson’s correlation coefficient (*r*) between AGB and individual UAV-derived vegetation indices and canopy height metrics. The underlined numbers represent statistical non-significance (*p* > 0.05)

Figure 4 shows a matrix of Pearson’s correlation coefficients (*r*) for the relationships between UAV-derived variables and their relationships with AGB. For the VI group, eight of the ten VIs showed highly positive or negative correlations with extreme *r* values up to 1 (GRVI vs. MGRVI, GLI vs. RGBVI, and GLI vs. ExG) or -1 (MGRVI vs. ExR and GRVI vs. ExR). VARI and ExB were the most strongly and weakly correlated to AGB, respectively (VARI: *r* = 0.79, *p*-value < 0.0001; ExB: *r* = 0.19, *p*-value < 0.005). For the height metric group, six of the eight metrics showed highly positive correlations with the maximum r value up to 1 (P50 vs. median, P50 vs. mean, median vs. mean, and std vs. cv). The highest correlations with AGB were found for P95 and P75 (*r* = 0.83, *p*-value < 0.05), with the lowest for cv (*r* = 0.07, *p*-value > 0.05). Generally, these correlations were stronger than those of VIs with AGB.

### Comparison of AGB estimation performance with the SMLR and machine learning techniques

Table 8 shows a comparison of the SMLR and three machine learning techniques for the estimation of AGB over the critical growth stages. Using the VIs alone, RF achieved best calibration (*R*^2^ = 0.70, RMSE = 1.51 t/ha, AIC = 369.23) and validation (*R*^2^ = 0.69, RMSE = 1.61 t/ha, rRMSE = 34.06%) performance among the three regression techniques, while the SMLR achieved the similar accuracy (validation: *R*^2^ = 0.70, RMSE = 1.58 t/ha, rRMSE = 34.49%). When using the canopy height metrics, the best performance was still found for RF and close performance for SVR and ELM. Compared with the SMLR, the performance of SVR and ELM was lower than the SMLR, while RF achieved the highest accuracy. Moreover, this accuracy for RF (Calibration: *R*^2^ = 0.73, RMSE = 1.44 t/ha, AIC = 398.32; validation: *R*^2^ = 0.74, RMSE = 1.39 t/ha, rRMSE = 30.95%) was even higher than that obtained using the VIs, with an increment of 0.05 in *R*^2^ and 0.22 t/ha in RMSE for the validation data.

**Table 8:** Accuracy assessment for the estimation of AGB in wheat from vegetation indices, plant height metrics and their combination with SMLR and three machine learning techniques

Input variables	Technique	Calibration (N = 201)			Validation (N = 85)		
		*R* ^2^	RMSE (t/ha)	AIC	*R* ^2^	RMSE (t/ha)	rRMSE (%)
VIs	SMLR	0.69	1.52	742.63	0.70	1.58	34.49
	SVR	0.69	1.53	696.27	0.64	1.75	38.13
	ELM	0.69	1.52	759.25	0.64	1.73	37.27
	RF	0.70	1.51	423.87	0.69	1.61	34.06
Canopy height metrics	SMLR	0.68	1.53	747.72	0.72	1.54	33.61
	SVR	0.68	1.56	708.20	0.70	1.55	33.71
	ELM	0.65	1.65	751.30	0.71	1.55	33.63
	RF	0.73	1.44	398.32	0.74	1.39	30.95
VIs and canopy height metrics	SMLR	0.72	1.43	717.41	0.75	1.46	31.77
	SVR	0.71	1.50	592.05	0.73	1.51	33.10
	ELM	0.72	1.45	733.02	0.73	1.51	32.77
	RF	*0.76*	*1.34*	*369.23*	*0.78*	*1.34*	*28.98*

The accuracy metrics were calculated from calibration data and validation data separately. The number in bold for each column represents the maximum *R*^2^, minimum RMSE, minimum AIC and minimum rRMSE, respectively

**Fig. 5:**
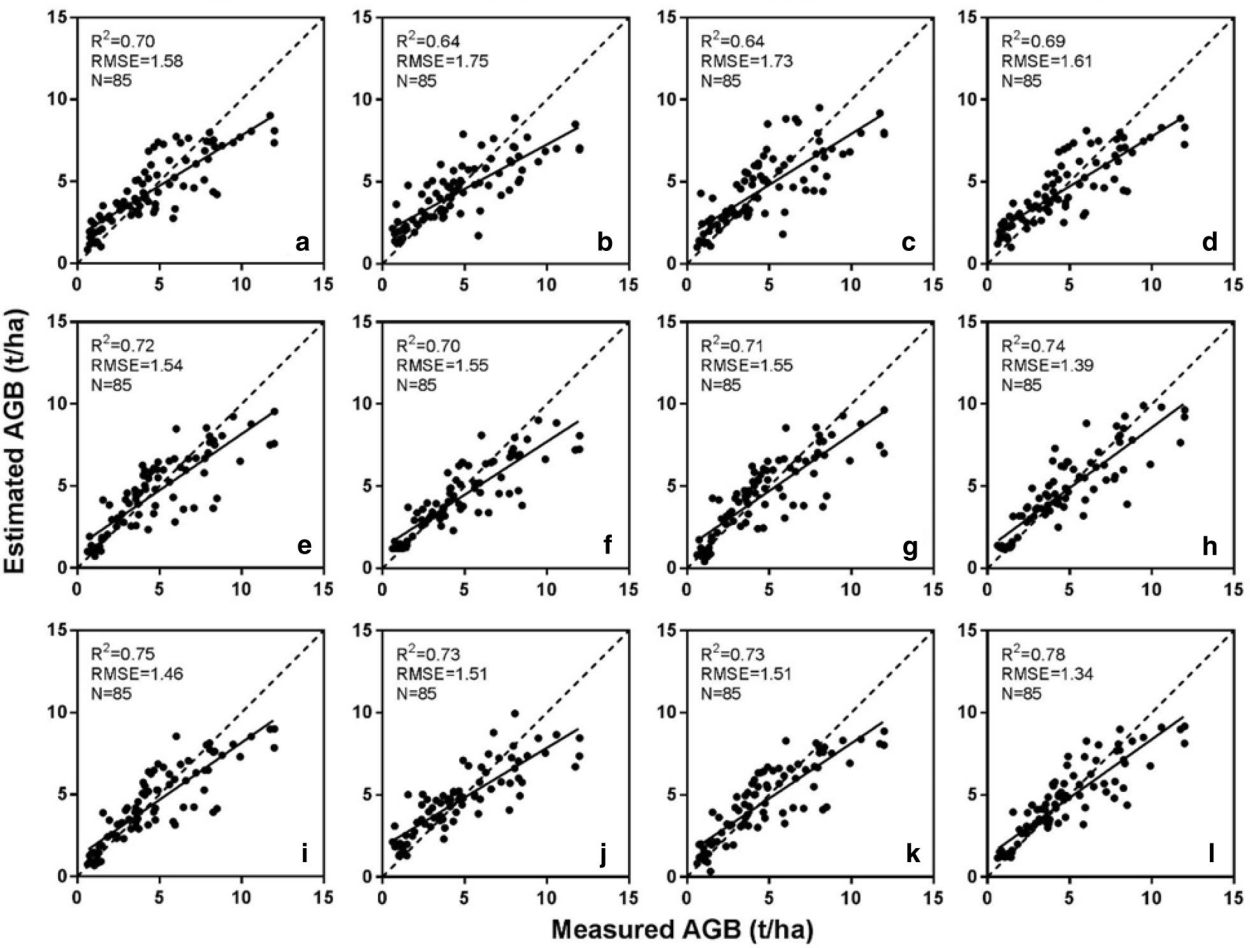
Scatterplots of measured AGB (t/ha) in wheat and the AGB estimated with three machine learning techniques (left: SMLR, second column: SVR, third column: ELM, right: RF) from the VIs alone (**a**–**d**), canopy height metrics alone (**e**–**h**) and the combined data (**i**–**l**). The data points displayed in each plot represent the validation set. The dashed diagonals represent 1:1 lines and the solid lines represent the fitted linear functions

The combination of VIs and canopy height metrics yielded further improvement for all regression techniques. Their accuracies were significantly higher than those achieved with the traditional approach of merely using VIs, with a uniform increment of 0.09 in validation *R*^2^ for all three regression techniques. Consistently, RF yielded the highest accuracy in calibration (*R*^2^ = 0.76, RMSE = 1.34 t/ha, AIC = 369.23) and validation (*R*^2^ = 0.78, RMSE = 1.34 t/ha, rRMSE = 28.98%). The scatter plots in Fig. 5 shows the data points are generally closer to the 1:1 line by combining the VIs and canopy height metrics but a small portion of them associated with high values of measured AGB are located under the diagonal.

### Performance for individual growth stages

**Fig. 6:**
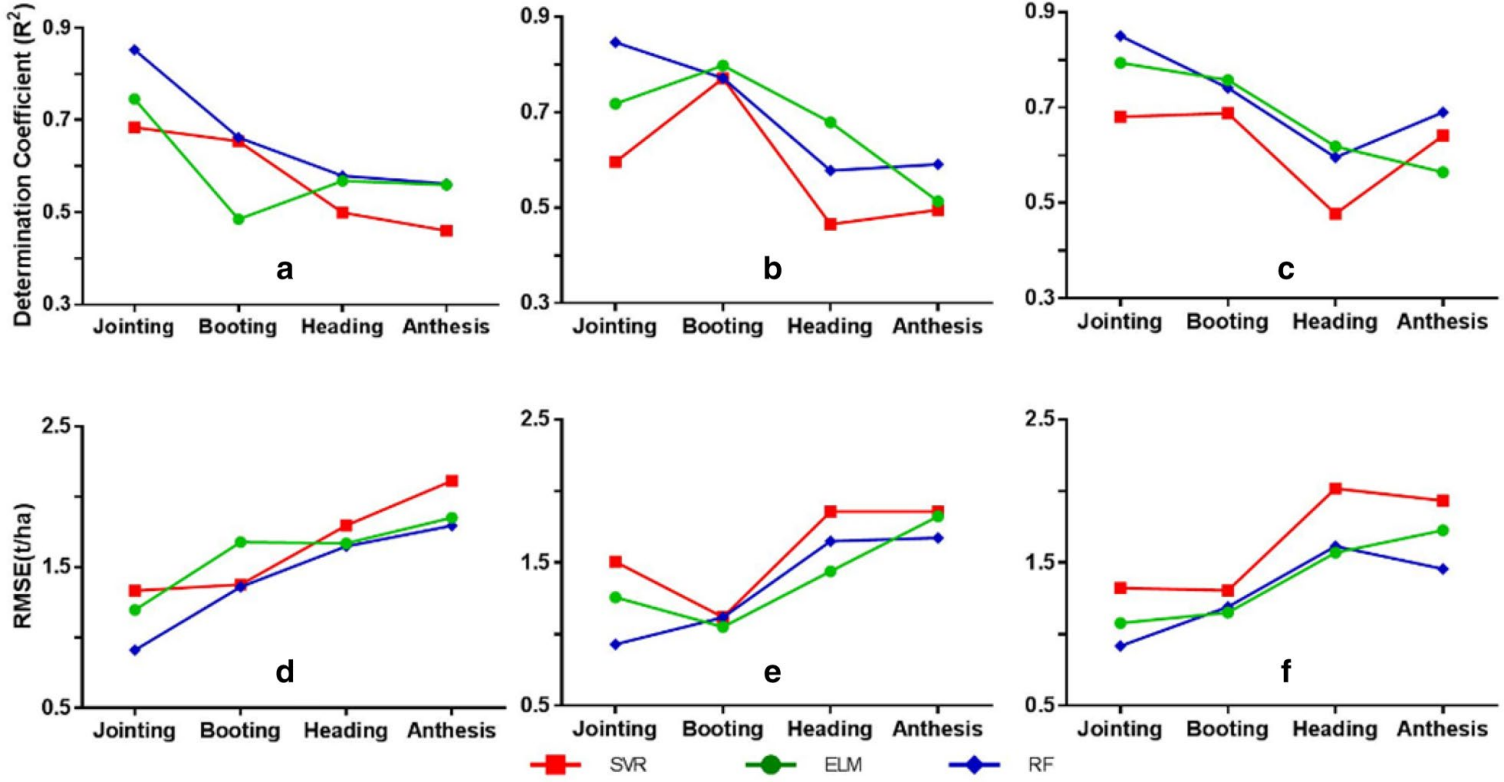
The performance of AGB estimation from three types of input data (left column: VIs; middle column: canopy height metrics; right column: combination of VIs and canopy height metrics) for individual critical growth stages with three machine learning techniques. The top and bottom rows represent validation assessment in *R*^2^ and RMSE, respectively

Figure 6 shows that the performance of AGB estimation was inconsistent across individual growth stages for the three types of input data and three regression techniques. The accuracy was generally a degradation trend from the highest to lowest for jointing to anthesis stage. The degradation of estimation accuracy from booting to heading was the most prominent change for all neighboring stages. In addition, the booting stage was stable for observing AGB for all three machine learning algorithms. Among the three regression techniques, RF was mostly the best performing one and SVR was the most sensitive to growth stage. In contrary to the multi-stage situation, using the VIs as the input data for RF yielded better accuracies than using the canopy height metrics. However, the combination of VIs and canopy height metrics consistently performed better than either type of input data alone. A similar change trend was observed when assessing the accuracies with RMSE.

### Effect of spatial resolution on AGB estimation

**Fig. 7:**
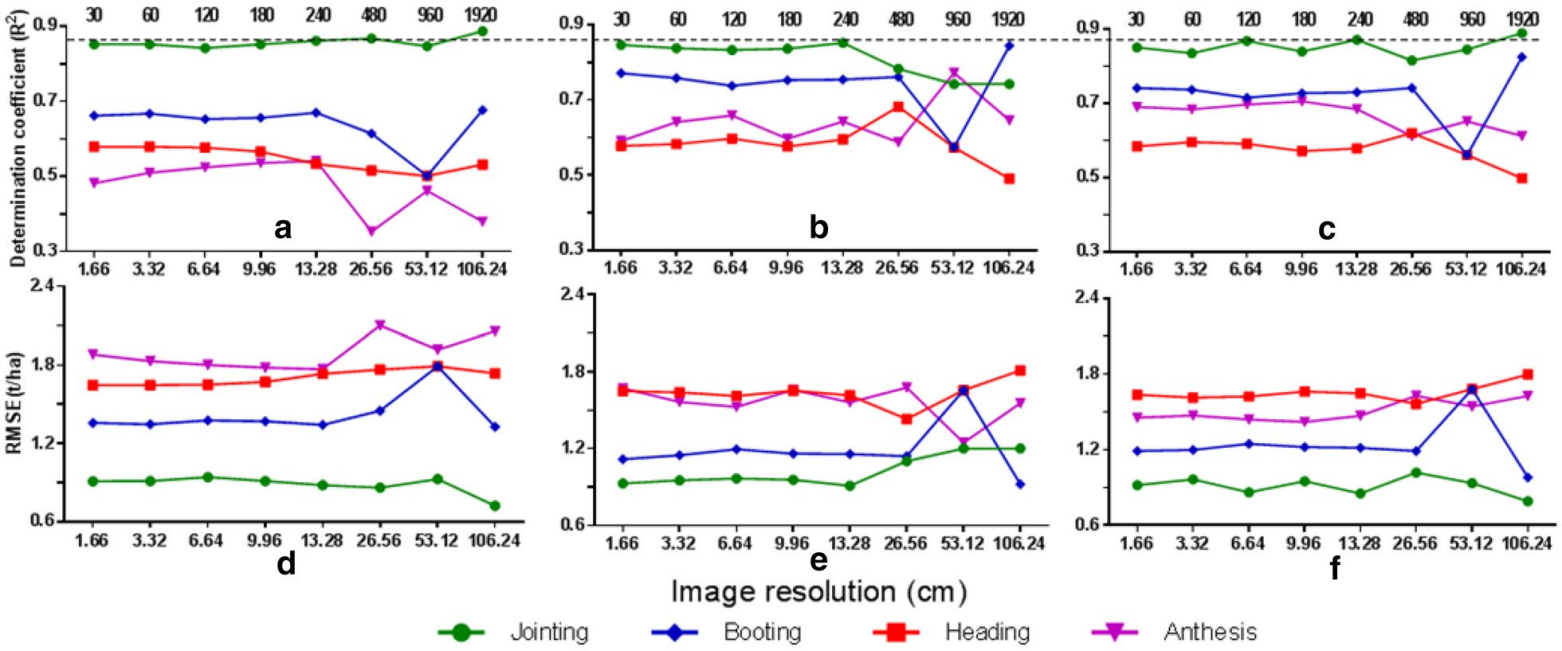
The performance of AGB estimation from three types of input data (left column: VIs; middle column: canopy height metrics; right column: combination of VIs and canopy height metrics) for a series of pixel sizes with the RF technique. The top and bottom rows represent validation assessment in *R*^2^ and RMSE, respectively. The top number represents the flight altitude of UAV, while the number label of x-axis is the image resolution consistent with flight altitude

The performance of AGB estimation for a number of image resolutions is displayed in Fig. 7. Generally, the accuracies were more variable for smaller pixel sizes and the comparable accuracy was obtained for 13.28 cm using canopy height metrics or the combined data as input for RF. Canopy height metrics performed better than VIs over the series of pixel sizes for AGB estimation at each critical growing stage. By combining the VIs and canopy height metrics, the performance was less sensitive to pixel size and their combination performed slightly better than the use of VIs and canopy height metrics alone.

## Discussion

### Comparison of SMLR and the machine learning techniques

SMLR is a commonly used method for selecting explanatory variables in multivariate regression and is prone to overfitting in quantifying vegetation parameters [34]. Our results demonstrated that the performance of SMLR was worse than that of RF and comparable to those of SVR and ELM, which was consistent with the findings in Li et al. [7]. The stable regression performance of SMLR was probably attributed to the relatively small number of input variables (no more than 18) as compared to hundreds or thousands of bands in spectroscopy analysis [58]. To evaluate its performance in variable selection, we tested the machine learning regression techniques with the variables selected by SMLR as the input data. The calibration and validation accuracies with selected variables (Table 9) were not consistently higher across three groups of input variables than those with all the original variables (Table 8), which means the variable selection did not help improve the regression performance significantly. In fact, variable selection for the machine learning algorithms would not only increase the complexity of data processing, but also bring the uncertainty due to the deficiency of high inter-correlated variables. Using all the 18 variables for the combined data would make a big burden since the machine learning algorithms are able to handle high-dimensional data. Since variable selection may be useful for reducing the data volume in case of large data sets, future research may include searching for advanced selection procedures other than SMLR.

**Table 9:** Accuracy assessment for the estimation of AGB in wheat from the selected input variables with SMLR and three machine learning techniques

Input variables	Technique	Calibration (N = 201)			Validation (N = 85)		
		*R* ^2^	RMSE (t/ha)	AIC	*R* ^2^	RMSE (t/ha)	rRMSE (%)
VIs	SMLR	0.69	1.52	742.63	0.70	1.58	34.49
	SVR	0.72	1.49	696.91	0.65	1.70	37.19
	ELM	0.68	1.56	758.14	0.68	1.64	35.77
	RF	0.70	1.49	438.29	0.64	1.73	36.93
Canopy height metrics	SMLR	0.68	1.53	747.72	0.72	1.54	33.61
	SVR	0.70	1.52	697.85	0.73	1.49	32.66
	ELM	0.69	1.50	747.92	0.72	1.54	33.72
	RF	0.72	1.46	431.34	0.76	1.40	30.76
VIs and canopy height metrics	SMLR	0.72	1.43	717.41	0.75	1.46	31.77
	SVR	0.74	1.38	679.36	0.73	1.51	33.07
	ELM	0.72	1.44	714.49	0.73	1.50	32.77
	RF	*0.76*	*1.33*	*375.06*	*0.78*	*1.34*	*29.31*

The input variables for each technique were selected with SMLR. The accuracy metrics were calculated from calibration data and validation data separately. The number in bold for each column represents the maximum *R*^2^, minimum RMSE, minimum AIC and minimum rRMSE, respectively

### The improvement from the combination of VIs and canopy height metrics

The use of VIs represents a widely used approach to estimating crop AGB from UAV images, but its performance remains to be improved, especially when only RGB images are available. The reasons for the lower accuracy obtained by using VIs alone can be attributed to three aspects. Firstly, the VIs were derived only from the RGB bands and the lack of near-infrared (NIR) bands precluded the enhancement of contrast in vegetation vigor [19, 59]. Secondly, VIs were prone to saturate in high biomass conditions [60]. Thirdly, VIs were directly calculated from digital number (DN) images and it was hard to convert DNs to reflectance due to the wide spectral ranges of visible bands and inaccurate spectral response functions [11]. Moreover, the spectral information in the VIs was mainly from the leaves or panicles in the top layer of wheat canopies. Since the AGB in wheat encompassed the biomass of leaves, stems and panicles, the VIs might not reflect the information from stems that have a higher proportion of AGB compared to leaves in the middle to late growing season.

Canopy height is an important metric for characterizing vertical structure. Previous studies have shown a moderate relationship between canopy height and biomass for barley [1], grassland [61], maize [7]. This was confirmed by the good performance of canopy height metrics in the current study, even though the CHM-derived plant height was slightly lower than the field measurements (Fig. 3). Similar underestimations were also reported by Bendig et al. [21]. The reasons for the underestimations could be explained in two aspects. Firstly, recurring wind in the field might blow the leaves in the canopy so that the position of the same leaves would change in overlapping images. Secondly, the top of a wheat plant was sharp and it was challenging to capture the canopy top at the spatial resolution of 1.66 cm in the UAV images.

The VIs and canopy height metrics used in this study were separately derived from orthophotos and CSMs, which were both generated with overlapping RGB images acquired from a consumer-grade UAV system. The orthophotos recorded canopy surface spectral properties in three visible bands [7], while the CSMs characterized canopy vertical structure [59, 62]. Combining VIs and canopy height metrics as the input data for regression techniques enabled the use of two types of information sources which are spectral information and structural information. Our results suggest that the use of combined information exhibited better performance for AGB estimation than the use of spectral or structural information alone. Li et al. [7] investigated the combination of spectral and structural information for AGB estimation in maize, but they did not provide an explicit comparison among the three types of input data. Their study only covered one growth stage in maize and did not consider the performance of the combined information for multiple growth stages. Our study used a combination of ten VIs and eight canopy height metrics to estimate wheat AGB for the critical growth stages for 2 years. Such a number of input variables for the regression techniques provided sufficient spectral information about the top canopy and the structural information about the canopy vertical gradient.

### The optimal spatial resolution for AGB estimation

This study used the RGB imagery acquired with a low-cost UAV system to estimate AGB and obtained a *R*^2^ up to 0.78 for the multiple growth stages with the RF regression technique. Such high resolution images (1.66 cm pixel^−1^) would have to be collected at low altitudes with that system, which may be a limiting factor for the efficiency of image collection over large areas [63]. This problem can be overcome by using a higher resolution camera or flying at a higher altitude. Nevertheless, a higher resolution camera may lead to the increase in cost and weight, which may shorten the UAV flight duration compared with a lightweight and consumer-grade camera. Therefore, the sensitivity of AGB estimation accuracy to image spatial resolution was an important reference for the configuration of a UAV flight altitude. As indicated in Zarco-Tejada et al. [63], a relatively lower image resolution may still yield an acceptable accuracy. Our results suggested that it was feasible to adjust the flight altitude and maintain comparable performance at the same time (Fig. 7).

**Fig. 8:**
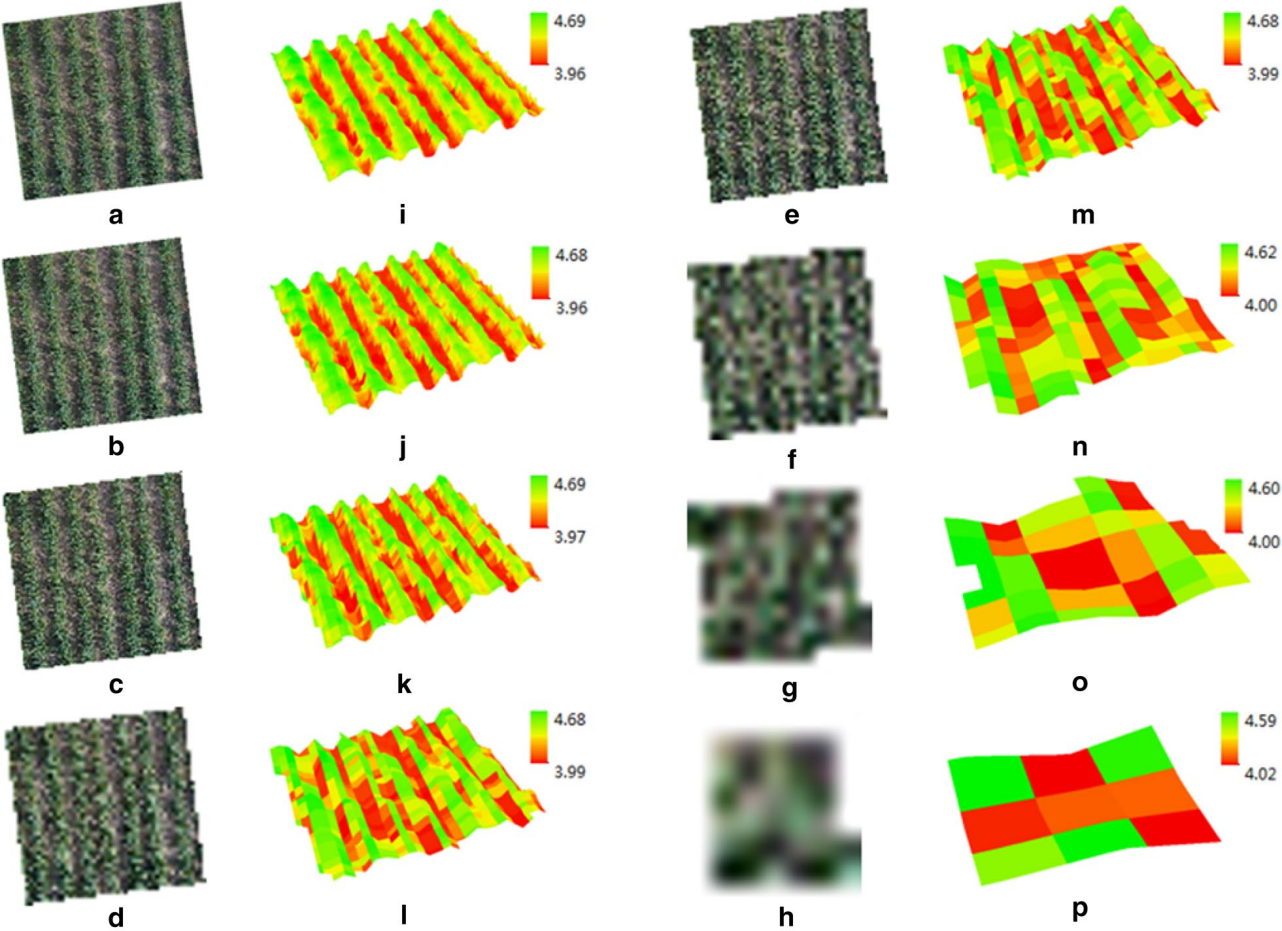
Spatial degradation of orthophotos from the original 1.66 cm pixel^−1^ resolution (**a**) to 3.32 cm pixel^−1^ (**b**), 6.64 cm pixel^−1^ (**c**), 9.96 cm pixel^−1^ (**d**), 13.28 cm pixel^−1^ (**e**), 26.56 cm pixel^−1^ (**f**), 53.12 cm pixel^−1^ (**g**) and 106.24 cm pixel^−1^ (**h**) used for the calculation of spectral vegetation indices. The corresponding digital surface models (DSMs) are displayed in **i**–**p**

The results demonstrated that the image resolution at 13.28 cm pixel^−1^ was the optimal for AGB estimation. Compared with original resolution of 1.66 cm pixel^−1^ acquired at 30 m, the shape of wheat canopy changed slightly but it was still easy to be identified at 13.28 cm pixel^−1^ (Fig. 8). However, when degrading to the lower resolutions, the mixed pixels from soil background and wheat led to the lower accuracy for AGB estimation (Fig. 7). Therefore, we suggest that UAV campaigns be carried out at 240 m to achieve the resolution of 13.28 cm pixel^−1^. That means we might be able to increase the efficiency to eight times with the same UAV system without a compromise of the estimation accuracy. The images at lower resolutions (e.g., 13.28 cm pixel^−1^) could be obtained by either increasing the flight altitude or using a lower-definition camera. Flying at 240 m is technically feasible and currently allowed by the local aviation regulation policy. Considering the difficulties in locating GCPs on the 13.28 cm pixel^−1^ image, one solution for future work would be to use new UAV systems with embedded RTK unit and avoid the use of GCPs.

### Comparison of RF to SVR and ELM for AGB estimation

Machine learning techniques have proved to be powerful for non-linear regression between remotely sensed data and biomass [64, 65]. This study evaluated the performance of three machine learning techniques with VIs, canopy height metrics and their combination as the input variables for AGB estimation, respectively. The results demonstrated that RF outperformed ELM and SVR consistently. In relevant studies on the estimation of crop AGB, RF was also found to be superior to SVR and artificial neural network (ANN) for wheat [7] and to stepwise multiple linear regression (SMLR) for maize [7, 12] and wetland vegetation [10].

RF regression is considered as one of popular ensemble learning algorithms for combining a large set of regression sub-models [66]. It is capable to model a large number of inter-correlated input variables and is not sensitive to noise or over-fitting [37, 67]. SVR tries to fit a hyperplane with calibration data as many as possible based on statistical learning principle. The estimate accuracy of SVR depends on a proper meta-parameters settings and selection of the kernel function. The optimal parameters can be obtained by grid search and iterative tuning. ELM is an efficient and rapid learning algorithm without much human intervention and does not need any kernel function. In this study, most of these variables derived from the UAV images were inter-correlated. RF is more suitable for dealing with two or more variables correlated with each other due to its insensitiveness to collinearity [12]. Previous studies have also proved that it is more likely to achieve high accuracy with RF due to its stability and robustness for complex and non-linear regressions [12, 64, 66]. The performance of RF for AGB estimation in wheat still needs to be validated with data sets from more study sites and varieties.

## Conclusions

This study compared the performance of the SMLR and three machine learning techniques for AGB estimation with VIs, canopy height metrics and their combination derived from high overlapping imagery acquired with a low-cost UAV system. Results demonstrated that the combination of VIs and canopy height metrics with all regression techniques improved the estimation accuracy over the use of VIs or canopy height metrics alone. In addition, RF yielded the most accurate estimations among the four regression techniques. Using RF, we demonstrated that a comparable accuracy for AGB estimation was obtained at the resolution of 13.28 cm pixel^−1^, which was reduced to one-eighth of the original orthophotos.

The findings imply that a consumer-grade camera mounted on a lightweight UAV could yield an accuracy of *R*^2^ up to 0.78 and a RMSE up to 1.34 t/ha for the AGB estimation in wheat. We proposed an inexpensive approach consisting of the RF algorithm and the combination of VIs and canopy height metrics derived from a low-cost UAV system at the consumer-grade level. This approach can be assessed for the efficient and economic monitoring of other growth parameters such as leaf area index in future research.
